# Healthcare providers’ perception of caring for older patients with depression and physical multimorbidity: insights from a focus group study

**DOI:** 10.1186/s12875-024-02447-9

**Published:** 2024-06-21

**Authors:** Laura Tops, Mei Lin Cromboom, Anouk Tans, Mieke Deschodt, Mathieu Vandenbulcke, Mieke Vermandere

**Affiliations:** 1 Department of Public Health and Primary Care, KU Leuven, Leuven, Belgium; 2 Competence Center of Nursing, University Hospitals Leuven, Leuven, Belgium; 3 Department of Neurosciences, Leuven Brain Institute, KU Leuven, Leuven, Belgium; 4 University Psychiatric Center, KU Leuven, Leuven, Belgium

**Keywords:** Multimorbidity, Collaborative care, Depressive disorder, Older adults, Multidisciplinary teams

## Abstract

**Background:**

The caretaking process for older adults with depression and physical multimorbidity is complex. Older patients with both psychiatric and physical illnesses require an integrated and comprehensive approach to effectively manage their care. This approach should address common risk factors, acknowledge the bidirectional relationship between somatic and mental health conditions, and integrate treatment strategies for both aspects. Furthermore, active engagement of healthcare providers in shaping new care processes is imperative for achieving sustainable change.

**Objective:**

To explore and understand the needs and expectations of healthcare providers (HCPs) concerning the care for older patients with depression and physical multimorbidity.

**Methods:**

Seventeen HCPs who work with the target group in primary and residential care participated in three focus group interviews. A constructivist Grounded Theory approach was applied. The results were analyzed using the QUAGOL guide.

**Results:**

Participants highlighted the importance of patient-centeredness, interprofessional collaboration, and shared decision-making in current healthcare practices. There is also a need to further emphasize the advantages and risks of technology in delivering care. Additionally, HCPs working with this target population should possess expertise in both psychiatric and somatic care to provide comprehensive care. Care should be organized proactively, anticipating needs rather than reacting to them. Healthcare providers, including a dedicated care manager, might consider collaborating, integrating their expertise instead of operating in isolation. Lastly, effective communication among HCPs, patients, and their families is crucial to ensure high-quality care delivery.

**Conclusion:**

The findings stress the importance of a comprehensive approach to caring for older adults dealing with depression and physical comorbidity. These insights will fuel the development of an integrated care model that caters to the needs of this population.

## Introduction

Persons with mental illnesses have a shorter lifespan than the general population, mostly due to physical comorbidities [1]. Having a mental illness almost doubles the risk of cardiovascular diseases, diabetes, and obesity in comparison to healthy persons [2, 3]. Moreover, compared to patients with chronic physical conditions, patients with mental illness also have higher rates of hospitalization and emergency department use [4, 5]. Among older individuals with depression, more than two-thirds present at least one somatic illness, and more than half of those with somatic comorbidities have two or more such illnesses [6]. Furthermore, older people with mental illnesses face the dual stigma of being both a geriatric and psychiatric patient [2].

Traditional care for older adults with mental illnesses lacks an integrated approach [7]. The effective management of their care requires a comprehensive approach that addresses common risk factors and the bidirectional relationship between somatic and mental health conditions, and integrates treatment for both [3, 8, 9]. The integration of mental and somatic healthcare is a top priority in national and international policy documents [2, 5, 6, 10–12].

A recent scoping review identified the intervention components that are commonly used within complex multicomponent care models for older adults dealing with both depression and physical multimorbidity [13]. Findings indicated that many of these care models share similar elements, such as the use of multidisciplinary teams, care coordinators, considering treatment interactions (e.g., polypharmacy, guideline interaction), continuity of care, individualized care planning, and personalized, holistic assessments with self-management support [13]. The findings of the review underscore the importance of recognizing the commonalities in intervention components within care models for older adults dealing with depression and physical multimorbidity. This understanding serves as a foundation for the subsequent discussion, which will delve into the practical aspects of implementing such interventions and the significance of stakeholder engagement in shaping their successful execution.

Bridging the gap between research and practice is crucial for the successful development and implementation of new healthcare interventions. Gathering valuable insights and perspectives on current practices from all relevant stakeholders (e.g. patients, informal caregivers, healthcare providers and policy makers) as part of a contextual analysis plays an essential role in ensuring the development of effective interventions aligned with their expertise and preferences. Incorporating implementation science principles further enhances the likelihood of successful adoption by addressing barriers and optimizing the implementation process [14]. Involving stakeholders in healthcare research also presents certain difficulties. For instance, when diverse individuals with their unique interests come together, it can result in complex situations, particularly when making decisions [15]. In healthcare research, stakeholder involvement can lead to the accumulation of different viewpoints and perceptions and increased trust and legitimacy among service users [16, 17], improving the quality, relevance and impact of health research [18, 19]. However, despite the direct influence changes in healthcare policy have on stakeholders, they are not always involved in the decision-making process [20]. Professionals’ practical experience grants them a deep understanding of specific contexts, allowing them to grasp nuances that may elude outsiders. Healthcare providers play a vital role in the realm of elderly care. In scholarly literature, they are recognized as mediators of context-specific knowledge, serving as invaluable conduits for insights tailored to the needs of older individuals [21, 22].

The focus group interviews within this study form an integral component of the context analysis conducted within the framework of the I-CONNECT project. Standing for ‘Integrated care program for home-dwelling older adults with depression and physical multimorbidity,’ I-CONNECT aims to comprehensively address the healthcare needs of this specific demographic. The results of the focus group interviews will fuel the next stages of the development of an integrated care model that caters to the needs of this population. Therefore, the objective of this study is to delve into the perspectives of healthcare providers concerning the provision of care for older adults facing both depression and physical multimorbidity.

## Methods

### Design and setting

Focus groups were the preferred method because of the possibility for interaction between participants. By bringing together individuals with diverse backgrounds and viewpoints, we aimed to create a dynamic environment for exchanging ideas and exploring multiple perspectives on the given topic. The focus group interviews were conducted at the University Psychiatric Centre (UPC-KU Leuven), a Belgian academic psychiatric hospital. The study complied with the Consolidated Criteria for Reporting Qualitative Research (COREQ) [23].

### Participants and recruitment

We conducted focus group interviews with HCPs who engage in regular professional interactions with older adults experiencing depression and physical comorbidities. To recruit participants, HCPs working in primary (e.g. home nursing, GP practice) and residential care (e.g. psychiatric hospital), and who have professional interactions with the target group, were contacted via e-mail or telephone and informed of the study’s aim. Flyers were also disseminated at strategic locations such as hospitals, doctor’s offices and pharmacies. Participants were given the opportunity to choose between an online or in-person format.

#### Eligibility criteria

Eligibility criteria were established to identify suitable participants in both residential and primary care settings. Within the residential setting, eligible participants included professionals holding the following professions: geriatric psychiatrist, geriatrician, nurse, and social worker. In the primary care setting, eligible participants encompassed general practitioners, psychologists, physiotherapists, pharmacists, and home care providers (e.g., domestic services, home nursing).

To be included in the study, participants had to be employed at the academic psychiatric hospital UPC KU Leuven or within the primary care vicinity of UPC KU Leuven. Participants were expected to have frequent professional interactions with patients aged 65 and above who presented with psychiatric and physical conditions. Proficiency in understanding and speaking the Dutch language was a prerequisite for inclusion.

We aimed for a focus group size of minimum six and maximum ten participants [24], which allowed everyone to share their opinion and also yield enough diverse information. Moreover, by not including too many participants, we created a safe environment where everyone was comfortable enough to express themselves freely [25].The researchers used a maximum variation purposive sampling based on gender, profession, and working experience to recruit participants, following the principles of Patton et al. [26]. Participants signed the informed consent form in duplicate and received a voucher of 25 euros after completing the focus group discussion.

### Data collection

The focus group interviews took place in the months of November 2022, December 2022 and March 2023. Three focus group interviews were conducted, of which two in person and one online session. The time span of each focus group was approximately one and a half hour. All focus groups were audio-recorded with the consent of the participants. The online focus group session was also video-recorded.

The focus groups were led by an experienced external moderator (AT), who was a member of the research team but held no affiliation with the psychiatric hospital. A semi-structured topic guide was used during the focus group interviews (Annex I). The topic guide was created based on an earlier literature review [27]. First, the moderator commenced the session by informing the participants of the underlying purpose behind the research. She additionally provided comprehensive insights into her professional background, thereby establishing her expertise in the field. Questions were asked about participants’ perceptions of the current care and their perspective on the future care for older adults with depression and physical multimorbidity. The moderator ensured that all voices were heard and that the discussion did not deviate much from the topic [28, 29]. Participants were also prompted to reflect on their own perspectives, facilitating a more comprehensive understanding of the data. Throughout the focus group discussions, the moderator posed supplementary questions designed to elicit participants’ viewpoints. This approach ensured that participants not only shared their ideas but also provided the rationale behind their viewpoints [30]. Two observers (LT & MC) were present to take notes on the progress of the conversation and on non-verbal communication. These notes were integrated into the result section.

### Data analysis

We used the constructivist Grounded theory approach introduced by Charmaz [28] to gain a better understanding of healthcare providers’ (HCPs) perceptions of the care provided for older adults with depression and physical multimorbidity. Charmaz’s constructivist grounded theory aims to understand social phenomena and subjective experiences. By actively engaging with participants, iteratively analyzing data, and reflecting on our own biases, we can generate insights grounded in the perspectives of the HCPs. Inclusivity of diverse voices allows us to capture the complexity of participants’ experiences within their social contexts, contributing to a more comprehensive understanding of the phenomena under investigation [31].

Conversations were typed out verbatim. Participants were pseudo-anonymized in the transcripts by assigning them numbers. Two researchers (LT & MC) carried out the analysis by means of the Qualitative Analysis Guide of Leuven (QUAGOL) [32], a practical guide rooted in the constant comparative method of the Grounded Theory Approach [24]. The QUAGOL method guides the researcher to a comprehensive view of the qualitative interview data. The first part of the method is described as ‘paper and pencil work’, which constitutes the preparatory stage before the coding process. During this stage, researchers thoroughly review the transcripts, craft narrative reports, and endeavor to formulate concepts and, ultimately, a conceptual framework from the data [32].

The second part consists of the actual coding through the use of dedicated software [32]. Two researchers (LT & MC) independently coded the data with ATLAS.ti Web software. LT and MC carefully analyzed the interview transcripts, identifying important concepts related to the care of older adults with depression and physical health issues. MV then reviewed and, if needed, refined the initial themes to ensure a thorough analysis of the data.

## Results

### Participants

The focus groups in this study comprised 4 to 8 participants each, with a total of 17 healthcare providers taking part. The first focus group was composed of a heterogeneous group of HCPs, while the second and third focus group interviews had less heterogeneous profiles. In Table 1, the gender distribution of all participants shows that the majority were female, comprising 65% of the total. During the first focus group discussion, one participant was absent (reason not reported), resulting in a total of eight instead of the intended nine participants.

**Table 1:** Participant characteristics

	Focus group 1 *n* = 8	Focus group 2 *n* = 4	Focus group 3 Online *n* = 5
**Professions**	Social worker; head nurse; resident geriatrics; supervising psychiatrist; team coordinator; psychologist	Nurse; social worker; home nurse	Pharmacist; general practitioner
**Sex**	M = 3 F = 5	M = 1 F = 3	M = 2 F = 3

### Healthcare providers’ perceptions

Throughout the focus group interviews, participants shared insights on various subjects, including patient-centeredness, interprofessional collaboration, shared decision-making, technology, capacity building, proactive care, and effective communication. Each of these topics will be examined in depth in the subsequent sections.

#### Patient-centeredness

Participants emphasized the importance of individualized care tailored to the unique needs and living situation of each patient. They highlighted the need to identify and address aspects of care that can be adjusted to improve patients’ quality of life.

*Also looking from the perspective of the patient as much as possible, like hearing how it’s going, how they’re experiencing it. If it’s still possible, continuing to give as much control as possible to the patient (Focus group 2, participant 2).*

Many of the participants felt that it is crucial for patients to maintain control over their own care process for as long as possible. They highlighted the role of the environment in enabling patients to stay in control in their own surroundings. The participants also stressed the importance of keeping patients well-informed about available care options to facilitate good decision-making.

*I often also find it important that patients are well informed, that they are able to make informed decisions, weigh the options, and that you then work together towards a goal and preferably in consultation with the system as much as possible, whatever that system may be. And that can also be good neighbors or other involved parties. So I think that network part is also really important (Focus group 1, participant 5).*

According to some participants, striking a balance between the patient’s preferences and the necessary medical interventions is challenging. Furthermore, one participant underscored that patients’ capacity to manage their condition evolves with the stage of the illness. For instance, individuals in remission from depression may exhibit different control dynamics compared to those in the acute phase of the condition.

*To what extent are you going to acknowledge and follow the wishes of a depressed patient. And to what extent are you going to push good care, that we consider good care. That’s really difficult (Focus group 1, participant 8).*

*I don’t think you can expect a patient who has major depression to actively take control of their own care (Focus group 1, participant 5).*

#### Interprofessional collaboration

Participants value teamwork among different healthcare providers when dealing with complex patients. Some participants suggest that this could result in better continuity of care.

*Collaboration between partners to work on continuity of care, that’s also a challenge but is part of good care (Focus group 1, participant 6).*

Several caregivers suggested that interdisciplinary patient meetings provide an effective forum for collaborating with all stakeholders involved in a patient’s care. These meetings allow for the planning of an ideal course of care and provide an opportunity to discuss and assign responsibilities, as well as to evaluate what is achievable for everyone involved.

*Care consultations with family members and possibly those who already, if home nursing care comes to the home, to gather them around the table and to just hear how it’s going, how is everyone’s capacity, what is needed to get that clear (Focus group 2, participant 2).*

According to the participants, the collaboration among different healthcare settings can be improved. More emphasis could be placed on holistic care, where somatic and psychiatric conditions are treated together. To that end, healthcare providers across different settings chould be encouraged to work collaboratively in order to enhance the quality of patient care.

*And there’s such a gap between them and they need to come together. And I find, I think that we can best offer complete care, total care if we can unite those two (Focus group 2, participant 1).*

Several participants proposed the idea of a “coordinator” or “responsible caregiver” as a potential solution to enhance continuity of care and address the issues related to care coordination.

*Because we notice that there are very often problems with care coordination. That people often come by the house, but that no one has really thought about how they relate to each other and that sometimes someone else has to come along to get the job done (Focus group 2, participant 2).*

*Yes, it would be much better if the nurse, whose patient is going to the short-stay center, that they can remain the nurse in charge, to be the intermediary instead of us having to turn to another organization to temporarily take over (Focus group 1, participant 7).*

#### Effective communication

According to the participants, there is still potential for improvement in the area of communication. To enhance clarity regarding care tasks and time schedules, communication must be improved not only among healthcare providers but also between healthcare providers and patients/families. As previously discussed, implementing a shared communication channel has the potential to enhance communication among all stakeholders involved.

*P4: Yes, the communication between the various care providers, both specialists and other care providers. So that the multidisciplinarity, that it can improve (Focus group 3, participant 4).*

*We’ve already had situations and that’s mainly about who’s washing the patient uhm, is that the home nurse, is that the family help, who is taking up the care. That is frequent, that is something that occurs very often and that is then sometimes lost sight of because one person thinks that the other is doing that (Focus group 2, participant 2).*

*P3: Also maybe not having a channel. P2: [No channel] P1: [Not really knowing, I think] P2: [Yes] P3: [I once witnessed someone who had a sort of notebook and so then the one caregiver writes in the notebook and indeed then the next one comes another day and can then see aha yes that’s what happened (Focus group 2).*

#### Shared decision-making

Healthcare providers agree that developing an appropriate care plan requires coordination between the patient, their network, and caregivers, Where all parties’ wishes and opinions are considered as much as possible. According to some participants, involving family members in care consultations can be highly beneficial as they can provide valuable insights into the patient’s situation. Healthcare providers also emphasize the importance of understanding the patient’s home situation to ensure better care.

*That you then work together towards a goal and preferably in consultation with the system as much as possible (Focus group 1, participant 5).*

*When admitted, there is always a system discussion and with elderly patients we try to make sure that an involved party is present as much as possible, a partner but certainly also children. Because we also know that we need them in that story (Focus group 2, participant 3).*

#### Integrating technology in patient care

Healthcare providers agree that integrating eHealth can benefit the future of patient care. Participants provided specific examples such as digital shared medical files, tablets, automatic pill dispensers, exercise robots, and video consultations. While many healthcare providers recognize the potential benefits of eHealth and digitization (e.g. time effectiveness), significant improvements are still needed to ensure proper functioning and efficiency. Participants remarked that some older generations may struggle to keep up with changing technologies, which can hinder progress in this field.

*P2: There is still room for improvement in file management. M: [Yes? ] P2: Especially in opening the file because everyone works with a different file management system (Focus group 2).*

*I think there are two sides to that because I’ve noticed that many elderly people are being left behind because they can’t keep up with the technology and aren’t able to request certain things that they are entitled to (Focus group 2, participant 2).*

*Video or consultations by video call, I won’t say are an equal alternative but can be complementary in treatment or a follow-up or uhm a care pathway in any case. I think that that could come more in the future or could be installed more (Focus group 1, participant 6).*

Various caregivers emphasize the need to be vigilant about the dangers of healthcare technology. For example, they believe it is important to update the digital record as if the patient is reading along. They also highlight the importance of maintaining human contact despite increasing digitalization.

*That’s why there are more and more calls to write your reports with the knowledge that the patient is reading along (Focus group 1, participant 8).*

*I definitely think that that [eHealth] can be implemented more frequently in the future. But we do need to keep focusing on human contact (Focus group 2, participant 1).*

#### Pro-active care

During the discussion, some participants highlighted the need for a greater emphasis on preventive care measures. They observed that current medical interventions are reactive, only taken when problems have already arisen or when conditions have deteriorated, leaving patients in a more critical state. To address this issue, they suggested that more attention could be given to early care planning, which could help prevent the need for more drastic or specialized interventions later on.

*While I sometimes think, if they would do that quicker, make that threshold a bit lower, that the response can be faster and that depression can also be resolved quicker, easier. Whether that’s the case, I don’t know of course, that’s my feeling (Focus group 1, participant 8).*

*P2: Actually, that healthcare proxy is already a good start to arrange everything in advance. That could easily be highlighted a bit more. M: [Yes, could be emphasized] P1: [So the preventive aspect, right] P2: That you no longer have to decide for the person, I hope. P4: [That they can decide for themselves] (Focus group 2).*

*Sometimes letting it drag on a bit too long, after which a sort of crisis arises or sort of, or deteriorating even further so that even more specialized care is then necessary (Focus group 1, participant 8).*

#### Capacity building

Some respondents noted a concerning lack of knowledge among healthcare providers. Specifically, they mentioned that some HCPs seem to be unaware of how to effectively treat patients with somatic and psychiatric concerns, leading them to refer these patients to other healthcare providers. Enhancing the provision of specific training to HCPs regarding psychiatric and somatic illnesses can offer a promising solution.

*P1: Yes, geriatric departments are like “yes, that is a psychiatric patient” and then. P2: [and then they come to us. And then we think, our nurses say we can’t handle that] (Focus group 2).*

*So uhm yes, what I also want for the future, in my view, is to give the staff some more training, to give them some more guidance (Focus group 2, participant 4).*

One key point was the challenge of sharing knowledge effectively within organizations, underscoring the need for improved dissemination strategies. Additionally, the importance of allocating more resources and time for thoughtful decision-making in caregiving settings was emphasized, highlighting the human-centric nature of the work. Furthermore, the focus group interviews acknowledged the multifaceted challenges in caregiving, such as staffing shortages and resource constraints, demonstrating the need for enhanced support and resource allocation within the field.

*Many organizations work with coordinators and such and the coordinators do have knowledge and disseminate it among their caregivers, for example, but that the people on the floor don’t (Focus group 1, participant 5).*

*Are there any specific growth opportunities for you in your department? [M] (…) More thorough, more people. That you can actually work in a more focused way and don’t have to make a decision too quickly or can tackle things more thoroughly. I mean, you’re working with people and not with things (Focus group 2, participant 1).*

*But when I go there and I see that there is understaffing, I also understand that they say: We’re already short of hands, do we now have to go spend an extra week in training, so I understand that as well. And then we run into the fact that there is a shortage in various areas I think, in terms of staff, time, finances (Focus group 1, participant 8).*

## Discussion

Our findings based on the three focus group interviews demonstrate that placing patients at the core of the care process and empowering them to retain control over their own care for as long as possible is crucial. It is imperative for healthcare providers to collaborate effectively to elevate the quality of patient care. Furthermore, it could be beneficial for patients and families to be regarded as equal partners in the decision-making process. Participants highlighted several areas where improvements can be made. Technological features (e.g. digital shared medical files, tablets, automatic pill dispensers, exercise robots, and video consultations) can play a vital role in enhancing the efficiency of care processes, making them more time-efficient. Care could also consider shifting towards a more proactive approach, rather than solely relying on reactive measures. Additionally, the participants conveyed a shared belief in the potential benefits of optimal care coordination facilitated by a dedicated care manager. To enhance the delivery of high-quality care, it may be advisable for healthcare providers to undergo comprehensive training covering both psychiatric and somatic domains. Finally, to increase clarity regarding care tasks and time schedules, it is essential to enhance communication not only among healthcare providers but also between healthcare providers and patients/families.

Participants emphasized the importance of patient-centered care and shared decision-making (SDM). Encouraging the active participation of older depressed patients has been proven to improve their adherence to psychotherapeutic interventions [33]. Moreover, SDM can lead to higher levels of patient satisfaction and increased feelings of autonomy and empowerment [34]. Participants additionally stressed the importance of involving family in decision-making processes. According to the SELFIE framework for multimorbidity, engaging informal caregivers in shared-decision making is a critical aspect of integrated care programs [35]. Nevertheless, involving informal caregivers in shared decision-making is not yet a common practice in healthcare. Although informal caregivers are sometimes asked for their opinion, they are often not included in decision-making processes alongside the patient and healthcare providers [36]. Moreover, there is a lack of evidence in how to successfully implement SDM in healthcare settings [37, 38]. In the future, researchers should acknowledge the vital role that shared decision-making plays in this context and aim to make it a fundamental part of integrated care models. Furthermore, researchers should actively engage patients in research endeavors and seek to understand their perspectives on concepts such as ‘patient-centeredness’ and ‘effective communication.

Participants emphasized the role of multidisciplinary care in managing mental and physical comorbidity. Integrated care is important for effectively managing complex health conditions that involve both mental and physical illnesses. This approach recognizes that these illnesses are interconnected and require coordinated attention from multiple care providers who communicate and collaborate effectively. Achieving integrated care requires a shift in our approach to service delivery, management, and funding, with a focus on the person rather than the disease. This aligns with current national and international policies to integrate mental and physical health care [2, 5, 12]. Additionally, to provide optimal care for older adults with depression and physical multimorbidity, healthcare providers should possess expertise in both psychiatric and somatic domains [39–41], as emphasized by the participants in the focus group sessions. Alongside specific knowledge, effective knowledge sharing among healthcare providers also proved to be a crucial aspect in the focus group interviews. Future integrated care models must recognize the intricate interplay between mental and physical health conditions. Healthcare providers involved in these interventions could benefit from undergoing comprehensive training covering both somatic and psychiatric domains to better address the needs of this specific population. Staff members, such as chief nurses, might consider undergoing training to enhance their ability to effectively impart knowledge to other personnel. However, it’s essential to acknowledge and address implementation barriers such as resource and time constraints, as well as workload and staffing issues, to ensure the successful adoption of such training initiatives.

During the discussion, the concept of a “coordinator” or a “responsible caregiver” was introduced as a promising approach to improving the continuity of care and tackling care coordination challenges. Case management in primary care can be more effective if its focus is on enhancing the capabilities and perceived social support of the beneficiaries [42]. As such, there is uncertainty about whether case management improves patient and service outcomes or reduces costs [43]. Future research should focus on understanding what works in case management interventions, who benefits from them, and how they can be more effective.

Technological advancements in mental health care have the potential to empower patients and promote greater autonomy in managing their mental health. Concrete examples of such advancements include online psychological interventions and remote monitoring of patients’ progress [44, 45]. In certain situations, the use of technology-facilitated healthcare can result in an improved quality of life, decreased feelings of isolation, and strengthened social networks [46]. Nonetheless, healthcare providers must consider the obstacles that may impede the implementation of eHealth among the older population. A recent review explored the barriers and facilitators of the use of technology-facilitated health care (eHealth) in older adults [47]. These barriers can include, for instance, a lack of experience or proficiency with eHealth or technology [48–51], a lack of confidence in using eHealth solutions [52], and limitations related to aging [47]. Throughout the course of the present study, participants highlighted the advantages offered by eHealth, while also acknowledging potential challenges that may arise, such as ensuring privacy protection, preserving personal connections, and addressing accessibility issues for older individuals with regards to technology. To ensure the delivery of high-quality care, future integrated care interventions could explore the potential of technological advancements, such as video consultations and shared communication platforms, while considering the unique vulnerabilities of older adults.

In our study, we adopted an inductive approach, allowing themes to organically surface from the data. Nevertheless, we also contemplate the potential merits of employing a deductive methodology, such as employing established frameworks like the Consolidated Framework for Implementation Research (CFIR) to discern prevalent barriers and facilitators within care processes. Subsequent investigations could delve into these avenues for additional insights [53].These findings of this study contribute to the existing literature by examining the perspectives of healthcare providers on the provision of care for older adults with depression and physical multimorbidity. Focus group interviews were an optimal choice for qualitative research due to the valuable group dynamics and interactions they facilitated [54]. However, the study also has several limitations. Firstly, the number of participants varied significantly between the three focus groups, with eight participants in the first group, and only four and five participants in the second and third groups, respectively. This may have resulted in less diverse perspectives and answers in the smaller groups. Unequal group sizes can influence the dynamics within the focus group. Larger groups may dominate the discussion, silencing quieter participants and hindering diverse viewpoints. Conversely, smaller groups may lack diversity and limit the depth of discussion. Additionally, although we attempted to include participants with heterogeneous profiles, the first focus group consisted solely of residential healthcare providers, whereas the second and third group included HCPs from the primary care environment. This may have influenced the dynamics and outcomes of the focus groups. Furthermore, while the use of an online format for the third focus group discussion provided flexibility, opinions on online focus groups vary and this format may have affected the quality of data collected. Finally, it’s worth noting that the demographic information we collected from participants was somewhat limited, focusing solely on their gender and profession. It could be beneficial to gather additional details, such as years of experience, to explore potential variations in perceptions, particularly between healthcare providers who are at the beginning of their careers and those with more experience.

## Conclusion

In conclusion, improving care for older adults dealing with depression and multimorbidity requires a significant shift. Placing the patient at the center of the care process and empowering them to take responsibility for their own care for as long as possible is crucial to achieving desirable healthcare outcomes. Collaborative efforts among diverse healthcare providers, facilitated by a dedicated care coordinator, are essential. Additionally, the focus groups emphasized the importance of involving patients and family members in care decisions. Integrating technological features, such as digital shared medical files, tablets, automatic pill dispensers, exercise robots, and video consultations, can significantly improve the efficiency and timeliness of care processes. Furthermore, it may be beneficial for healthcare providers to receive comprehensive training in both somatic and psychiatric domains to effectively address the needs of this specific population, including training for staff members like chief nurses in knowledge sharing. There is a pressing need for improvement in communication, particularly among healthcare providers and between healthcare providers and patients/families, particularly with a view to enhancing clarity regarding care tasks and time schedules. By integrating these enhancements into future care models, we can ensure comprehensive and holistic care that addresses the unique needs of older adults with depression and physical multimorbidity.

## Annex

Annex I: Semi-structured topic guide.

1. **Patient persona (poster)**.

- What is the current state of care for Antoon?
- What are the key areas of concern for Antoon? E.g. medication interactions, fall prevention, adapted nutrition.
- What are your experiences with providing care for these patients?
- According to you, what is needed to deliver quality care to this target group? E.g. involving caregivers/family, evidence-based practice, etc.
- What aspects are going well?
- Are there any areas that need improvement?
- How would you describe the core values of care as currently organized? E.g. multidisciplinary care, shared decision making, person-centered, empathetic, etc.

2. **How would you shape the future of care?**

- What areas do you see as having potential for growth?
- What factors can contribute to better healthcare delivery?
- What can you do yourselves?
- What is the role of patients and their family/caregivers? Describe the ideal caregiver from your perspective.
- What is the role of the healthcare provider? How does the role of one provider differ from another?
- What are the core values or key issues that should be addressed? E.g. multidisciplinary care, shared decision making, person-centered, empathetic, eHealth, person-centered care, continuity of care, self-management, proactive care, etc.

## Data Availability

The data used and analyzed during the current study are available from the corresponding author on reasonable request.

## Abbreviations

COREQ: Consolidated Criteria for Reporting Qualitative Research
HCP: healthcare providers
I-CONNECT: Integrated care program for home-dwelling older adults with depression and physical multimorbidity
QUAGOL: Qualitative Analysis Guide of Leuven
UPC: University Psychiatric Centre
